# Oropouche Virus Exposure in Febrile Patients during Chikungunya Virus Introduction in the State of Amapá, Amazon Region, Brazil

**DOI:** 10.3390/pathogens13060469

**Published:** 2024-06-03

**Authors:** Raquel Curtinhas de Lima, Helver Gonçalves Dias, Thiara Manuele Alves de Souza, Débora Familiar-Macedo, Edcelha D’Athaide Ribeiro, Valmir Corrêa e Corrêa, Alex Pauvolid-Corrêa, Elzinandes Leal de Azeredo, Flávia Barreto dos Santos

**Affiliations:** 1 Laboratório das Interações Vírus-Hospedeiros, Instituto Oswaldo Cruz, Fundação Oswaldo Cruz (Fiocruz), Rio de Janeiro 21040-900, Brazil; raquellima@aluno.fiocruz.br (R.C.d.L.); helvergd@gmail.com (H.G.D.); thiara.biomed@gmail.com (T.M.A.d.S.); deborafamiliar@gmail.com (D.F.-M.); naideazeredo@gmail.com (E.L.d.A.); 2 Laboratório Central/Amapa (LACEN/AP), Macapá 68908-530, Brazil; edcelha1980@gmail.com; 3 Laboratório de Fronteira (LAFRON), Oiapoque 69980-000, Brazil; biomedicocorrea@gmail.com; 4 Laboratório de Virologia Veterinária de Viçosa, Departamento de Veterinária, Universidade Federal de Viçosa (UFV), Viçosa 36570-900, Brazil; pauvolid-correa@ufv.br

**Keywords:** surveillance, PRNT, arbovirus, RT-qPCR

## Abstract

*Oropouche orthobunyavirus* (OROV) is an arbovirus transmitted by midges that has been involved in outbreaks throughout Central and South America. In Brazil, human cases have been historically concentrated in the northern region of the country. Oropouche fever in humans range from mild clinical signs to rare neurological events, and is considered a neglected tropical disease in Brazil. Due to the clinical similarities to other arboviruses, such as chikungunya and dengue viruses, OROV infections are likely to be underreported. Chikungunya virus (CHIKV) cases in Brazil were first recognized in 2014 in the states of Amapá and Bahia in the north and northeast regions, respectively. Both OROV and CHIKV cause nonspecific symptoms, making clinical diagnosis difficult in a scenario of arbovirus cocirculation. Aiming to investigate OROV transmission during the CHIKV introduction in the state of Amapá located in the Brazilian Amazon, we conducted a retrospective molecular (RT-qPCR) and serological investigation in febrile cases (N = 166) collected between August 2014 and May 2015. All acute serum samples were negative for OROV RNA using RT-qPCR. However, neutralizing antibodies for OROV were detected using a plaque reduction neutralization test (PRNT_90_) in 10.24% (17/166) of the patients, with neutralizing antibody titers ranging from 20 to ≥640, suggesting the previous exposure of patients to OROV. Regarding CHIKV, recent exposure was confirmed by the detection of CHIKV RNA in 20.25% (33/163) of the patients and by the detection of anti-CHIKV IgM in 28.57% (44/154) of the patients. The additional detection of anti-CHIKV IgG in 12.58% (19/151) of the febrile patients suggests that some individuals had been previously exposed to CHIKV. Whether the OROV exposure reported here occurred prior or during the CHIKV circulation in Amapá, is unknown, but because those arboviral infections share similar clinical signs and symptoms, a silent circulation of enzootic arboviruses during the introduction of exotic arboviruses may occur, and highlights the importance of syndromic cases’ surveillance to arboviruses in Brazil.

## 1. Introduction

Emerging and reemerging arboviral diseases are a global public health problem. Brazil is a hotspot for enzootic and epidemic arboviruses. During the last centuries, several yellow fever outbreaks ravaged through large Brazilian cities [1,2]. Since the introduction of dengue virus (*Orthoflavivirus denguei*, DENV), in the 1980s [3], Brazil has been facing an hyperepidemic scenario, with all four DENV serotypes circulating and millions of cases reported overtime [4]. In the last decade, chikungunya virus (*Chikungunya virus*, CHIKV) and Zika virus (*Orthoflavivirus zikaense*, ZIKV) were also introduced and quickly spread across the country, causing major epidemics [5,6].

CHIKV is a single-stranded RNA alphavirus that was first isolated in the 1950s in Tanzania [7]. Three CHIKV genotypes have been identified so far, including the West African, the East–Central–South Africa (ECSA), and the Asian genotype. The West African genotype is believed to circulate in enzootic cycles in Africa, and the two others are believed to circulate in urban cycles around the world [8], including Brazil. As with other arboviruses, CHIKV infection results in specific and persistent immune responses against reinfection [9]. 

In Brazil, autochthonous CHIKV infections were first reported in the state of Amapá, located in the Amazon region, and in the state of Bahia, located in the northeast region of the country. Phylogenetical analysis suggests that CHIKV was introduced in Brazil by two different events, both in 2014 [6]. Ever since, CHIKV has caused several outbreaks all over the country, registering 1.5 million cases by 2022 [10]. Clinical manifestations in typical acute CHIKV infection are fever, arthralgia, myalgia, nausea, and vomiting, among others [11,12]. Arthralgia is the most common and prominent symptom reported by patients with chikungunya fever [11,12]. CHIKV clinical infection can also have atypical manifestations, affecting cardiological, neurological, and respiratory systems, and may result in death [13,14]. As for other arboviruses, asymptomatic infections have also been reported in percentages that vary according to the population studied [15,16], highlighting the complexity of clinical diagnosis in arbovirus infection.

Oropouche virus (*Oropouche orthobunyavirus*, OROV) was first described in Trinidad and Tobago in 1955 [17]. OROV genome consists of three segments of negative-sense and single-stranded RNA, which can lead to reassortment events [18]. In Brazil, OROV was first isolated in the 1960’s from the blood of pale-throated sloth (*Bradypus tridactylus*) in the state of Pará in the north region of the country [19]. Since then, multiple outbreaks have been reported in the region, where OROV apparently acquired an endemic profile. Despite most cases are reported in the Amazon region, clinical infection by OROV has been reported in other regions of the country [20,21]. Disease caused by OROV in Brazil is similar to those caused by other arboviruses, including chikungunya, such as fever, headache, arthralgia, myalgia, nausea, and vomiting [22]. 

Despite causing recurrent outbreaks in urban areas and being considered a candidate for the next epidemic across the Americas, OROV is still a neglected arbovirus. Recently, a new outbreak of oropouche fever has been reported in northern Brazil [23], and in 2023, an increased number of cases was observed, with 773 confirmed cases. In the first two months of 2024 alone, more than two thousand cases of oropouche fever have been reported across different states in the Amazon region [24].

As arboviral infections are often asymptomatic or cause nonspecific manifestations, clinical diagnosis is challenging. Moreover, due to the clinical similarities to other arboviruses, OROV infections are likely to be misdiagnosed or underreported. Therefore, epidemiological surveillance is essential to better understand transmission dynamics and develop future control measures. Here, we aimed to investigate OROV circulation during the first chikungunya fever epidemic in Brazil.

## 2. Materials and Methods

### 2.1. Study Site and Samples Collection

In this retrospective study, serum samples from 166 patients with clinical signs and symptoms compatible with arbovirus infection were collected between August 2014 and May 2015, during the first epidemic of chikungunya fever in Brazil. Samples were randomly selected for analysis and were representative of seven cities throughout the state of Amapá, including Oiapoque (N = 85), Macapá (N = 57), Porto Grande (N = 6), Santana (N = 9), Serra do Navio (N = 7), Tartarugalzinho (N = 1), and Laranjal do Jari (N = 1). Patients consisted of 93 females (56.02%) and 73 males (43.98%), and the age distribution consisted of 36 patients aged 1–18 years (21.69%), 107 patients aged 19–59 years (64.46%), 11 patients aged 60–88 years (6.63%) and, in 12 (7.23%) patients, age information was unavailable. In this retrospective study, some samples were not subjected to all tests due to insufficient volume.

The Brazilian Amazon region covers 5 million square kilometers and 9 different states, including the states of Acre, Amapá, Amazonas, Pará, Rondônia, Roraima, Tocantins, and Mato Grosso e Maranhão [25]. The state of Amapá is located in the extreme north of Brazil, with an area of 142,470.762 km^2^ and a demographic density of 5.15 inhabitants/km^2^ [26]. Amapá shares international borders with French Guiana (Oiapoque) and Suriname (Laranjal do Jari) to the north and to the west, respectively. Its eastern side is bathed by the Atlantic Ocean, and national borders are shared with the state of Pará to the south, the Amazon River to the southeast, and again the state of Pará to the west. The Amazon rainforest is the most predominant biome, and its capital is the city of Macapá [27].

### 2.2. Molecular Investigation of CHIKV and OROV 

Total RNA was extracted from serum samples of patients using the QIAamp Viral RNA Mini kit (Qiagen, Hilden, Germany), following the manufacturer’s protocol and stored at −70 °C. Real-Time Reverse Transcriptase Polymerase Chain Reaction (RT-qPCR) for CHIKV detection was performed using primers CHIKV 874 5′ AAAGGGCAAACTCAGCTTCAC 3′ and CHIKV 961 5′ GCCTGGGCTCATCGTTATTC 3′ and probe CHIKV 899-FAM§ 5′ CGCTGTGATACAGTGGTTTCGTGTG 3′, targeting NSP1 coding sequence, according to Lanciotti et al. [28]. OROV detection was conducted by a duplex protocol that also detects mayaro virus (*Mayaro virus*, MAYV). Primers OROV_FNF 5′ TCCGGAGGCAGCATATGTG 3′, OROV_FNR 5′ ACAACACCAGCATTGAGCACTT 3′ and probe OROV_FNP 5′(FAM) CATTTGAAGCTAGATACGG 3′ targeted S fragment coding sequence of OROV. MAYV primers and probes were MAYV_FNF 5′ CACGGACMTTTTGCCTTCA 3′, MAYV_FNR 5′ AGACTGCCACCTCTGCTKGAG 3′ and 5′(VIC) ACAGATCAGACATGCAGG 3′, targeting NSP1 coding sequence, all designed by Naveca et al. [29]. 

### 2.3. Serological Investigation of CHIKV

Enzyme immunoassays (ELISA) for detection of anti-CHIKV IgM (Euroimmun, Lübeck, Germany) and anti-CHIKV IgG (Euroimmun) were used for serological diagnosis of CHIKV exposure, according to manufacturer’s instructions. Briefly, serum samples were diluted 1:10 using diluent provided by the kit and added to microtiter wells coated with recombinant CHIKV antigens. After 1 h incubation at 37 °C, the wells were washed 3 times and peroxidase-labeled anti-human IgM or IgG were added, followed by 30 min incubation at room temperature (RT). Following this, the wells were washed again 3 times, chromogen-substrate solution (tetramethylbenzidine [TMB] plus hydrogen peroxide) was added and, after 15 min reaction at RT, the reaction was stopped with the addition of 0.5 M sulfuric acid. Optical densities (OD) were measured at 450 nm and 620 nm using a spectrophotometer reader. Index values were calculated by dividing sample OD using the kit-supplied calibrator. Results were interpreted as recommended by the manufacturer: <0.8 negative, ≥0.8 to <1.1 borderline, and ≥1.1 positive.

### 2.4. Neutralizing Antibodies Investigation of OROV

A plaque reduction neutralization test (PRNT) was used to detect the neutralizing antibodies (NAb) to OROV. Before the assay, serum samples were heated at 56 °C for 30 min to inactivate complement system proteins. Briefly, inactivated samples were screened using PRNT_90_ for OROV (strain BeAn 19991, GenBank accession #KP052852.1, #KP052851.1, #KP052850.1) at a single 1:20 dilution. Reactive samples to OROV were tested in two-fold serial dilutions that ranged from 1:20 up to 1:640 for their ability to neutralize plaque formation with OROV. For both assays, VERO cells were seeded on a density of 2 × 10^5^ cells/well in 6-well plates with Medium 199 (Invitrogen, Waltham, MA, USA) supplemented with 10% Fetal Bovine Serum (Invitrogen), 100 U/mL penicillin (Invitrogen), 0.1 mg/mL streptomycin (Invitrogen), and 0.025 µg/mL amphotericin B (Invitrogen), and were maintained at 37 °C in 5% CO_2_. The diluted samples were incubated in equal volumes with OROV for 1 h at 37 °C in 5% CO_2_. After incubation, the respective mix of OROV and diluted serum sample was transferred to the VERO cells monolayers and incubated for 1 h at 37 °C in 5% CO_2_ for adsorption. Lastly, the overlay solution containing 199 medium and 1% agarose was carefully added to the monolayers and it was once again incubated for 48 h. Cells were fixed with 8% formaldehyde for two more days, and washed and stained with 1% crystal violet solution for 1 h. Plaque-forming units (PFU) were counted using a white-light transilluminator. Serum samples were considered seropositive to OROV when it reduced at least 90% of the formation of viral plaques at least at a 1:20 dilution.

## 3. Results

### Oropouche Surveillance in Febrile Patients during Chikungunya Outbreak

Due to the outbreak during the period, we initially evaluated the febrile patients for current CHIKV infection. Molecular differential diagnosis was performed, and 20.25% (33/163) were positive for CHIKV. As expected, most cases were from exanthematic patients. Positive patients were mostly from Oiapoque (81.82%, 27/33), followed by Macapá (15.15%, 5/33) and Porto Grande (3.03%, 1/33). Recent CHIKV infection was also confirmed by the detection of specific IgM in 28.57% (44/154) of the cases, distributed among the city of Macapá (45,45%, 20/44), Oiapoque (38.64%, 17/44), Porto Grande and Santana (6.82%, 3/44), and Serra Navio (2.27%, 1/44). Previous exposure was demonstrated by the detection of anti-CHIKV IgG in 12.58% (19/151) of the cases. Once again, Macapá and Oiapoque had the highest number of positivity 36.84% (7/19) each, followed by Santana and Porto Grande (15.79%, 3/19 and 10.53%, 2/19, respectively). Overall, 48.19% (80/166) of the cases were positive for CHIKV in RT-qPCR, anti-CHIKV IgM and/or anti-CHIKV IgG.

Considering that OROV is an endemic virus in the Amazon region, we investigated the possibility of OROV cocirculation during the first chikungunya epidemic. All cases tested were negative for OROV RNA using RT-qPCR, Table 1. The RT-qPCR protocol used is a duplex that also detects MAYV infection; however, all cases tested were also negative for MAYV acute infection as well. 

Evidence of OROV exposure by the detection of NAb using PRNT_90_ was observed in 10.24% (17/166) of the patients, and 13 of them had Nab titers ranging from 20 to ≥640 (Table 1). Four cases were not subjected to serial dilutions for titers determination because there was not enough sample volume. 

**Figure 1:**
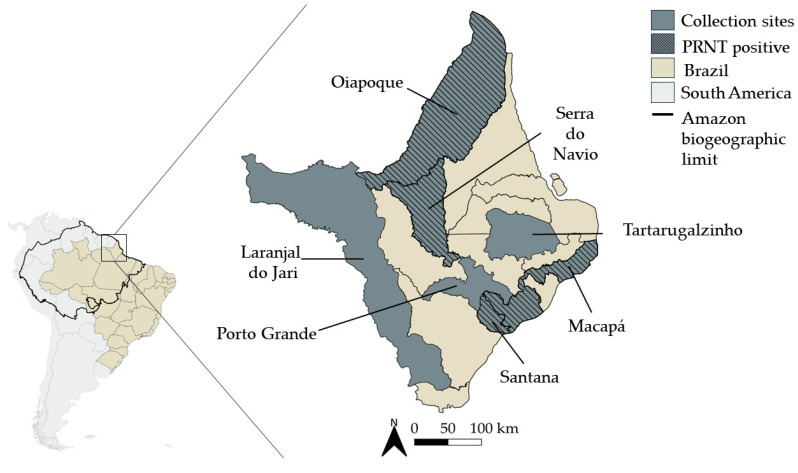
Distribution of oropouche-positive cases by PRNT_90_ in Amapá identified during a chikungunya fever outbreak, 2014–2015. Dark grey represents the collection sites and black lines represent the collection sites where oropouche-neutralizing antibodies were detected. For map construction, shapefiles were extracted from continuous Cartographic Base of Brazil, scale 1:250,000—BC250: version 2019 (Brazilian Institute of Geography and Statistics—IBGE) [30], and from Amazon Network of Georeferenced Socio-Environmental Information (RAISG) (2012) [31], prepared in QGIS 3.22.7 software.

Four cases were negative in all CHIKV tests and positive in PRNT_90_, three of them had an endpoint titer of 80, and one had an endpoint titer of 160 (Table 2). The patients were three females aged 56, 38, and 88 years old, and one 37 years old male from Macapá (1), Oiapoque (2), and Santana (1) (Figure 1).

## 4. Discussion

The introduction and rapid spread of CHIKV in the Americas show the expansive potential of arboviruses. Ten years after the virus’ introduction, more than three million cases, suspected and laboratory confirmed, were registered in 50 countries or territories in the Americas. Brazil has become an epicenter of chikungunya epidemic, reporting almost half of all these cases and registering annual epidemics [32]. 

Phylogenetic analysis indicate that Asian genotype was already in the Caribbean Islands 7 to 12 months before its detection in December 2013 [33]. The virus was possibly introduced several times through Brazil’ border with French Guiana in the second semester of 2014 due to intense migratory movements. The ECSA genotype had an independent introduction in Bahia, originating from Angola in the same year [6,33]. The cases presented here were collected during the first CHIKV epidemic in Brazil, which occurred in the state of Amapá in 2014–2015, and 48.19% (80/166) of the samples analyzed were positive for CHIKV with at least one of the tests used. Between April and September 2014, there was 23.5% positivity in Amapá [6]. Considering our time of collections between August 2014 and May 2015, the number of cases increased significatively; even so, 51.81% (86/166) of the cases were negative for CHIKV infection or past exposure.

Before CHIKV and ZIKV emergence, OROV was the second arbovirus with the highest number of cases in Brazil, surpassed only by DENV [34]. Although its first isolation in Trinidad and Tobago in 1955, phylogenetic analysis indicated that OROV emerged in Brazil approximately 223 years ago [35]. Several outbreaks and epidemics were reported in Peru, Panama, Trinidad and Tobago, and Brazil, and it is estimated that more than half a million people have already been infected at some point by OROV in the country [36]. Moreover, to date, four genotypes have been described, and all of them circulate in Brazil [36].

Taking into account the endemic nature of OROV in the Amazon region, the detection of CHIKV infection in the same region represents a challenge for public health in Brazil. Despite different vectors and epidemic patterns, oropouche and chikungunya fevers present similar clinical signs/symptoms, which can be particularly challenging for clinical and epidemiological diagnosis. The silent circulation of OROV during CHIKV introduction in Northern South America was corroborated by studies conducted in Peru and Brazil [20,37,38,39]. In 2016, 17% of 268 Peruvian acute febrile syndrome cases were OROV positive using RT-qPCR [37]. In Brazil, OROV has been recently detected in cerebrospinal fluid and serum samples of patients from Western Brazilian Amazon, and in an outbreak of febrile illness in the state of Bahia in the northeast region of Brazil [38,40]. In the last decade, evidence of OROV circulation in Brazil has also been reported in pools of mosquitoes [20] and in non-human primates [20,39]. 

Even testing human samples with a molecular protocol widely used by the network of Public Health Laboratories in Brazil [29,41], no acute cases of OROV infection were confirmed in the present study. One factor to consider is that molecular diagnosis of OROV is recommended to be performed up to seven days after the onset of symptoms [41]. The recurrence of symptoms observed during oropouche fever may influence the perception of the onset of symptoms reported by some patients misleading the right timing for RT-qPCR testing [42].

Serological investigation described here identified 10.24% of the patients with neutralizing antibodies for OROV, indicating viral circulation in the state of Amapá. The first confirmed outbreak of OROV in the country occurred in 1961, when OROV was isolated from the blood of residents of the urban area of Belém in the state of Pará. All patients presented symptoms such as headache, fever, and joint and muscle pain [19]. Furthermore, 27.78% of the samples collected for serological survey were positive in the neutralization test at the time of first collection [19]. In 1975, in Mojuí dos Campos, more than one thousand kilometers away from the capital Belém, the virus was isolated from 42 people and from pools of the midge *Culicoides paraensis*. A hemagglutination inhibition test also showed that 40% of students aged 18 years old and younger had anti-OROV antibodies [43]. Evidence of OROV circulation in the city of Manaus in the state of Amazonas has also been identified through the detection of anti-OROV IgM antibodies in patients with 5 or more days of acute febrile illness and negative for DENV and malaria. Of the 631 patients, 20.3% had already been exposed to OROV [44]. 

The genus *Orthobunyaviridae* is divided into serogroups according to antigenic relationships and genetic characteristics [18]. In the present study, serum samples were tested using PRNT_90_ only for OROV, which is part of the Simbu serogroup, along with jatobal virus and utinga viruses [45,46]. Despite cross-reactivity of some bunyaviruses have been reported mainly among viruses within the same serogroup, cross-reactivity cannot be fully discarded [47,48]. Aiming to mitigate this possibility, we investigated OROV, which is the most common orthobunyavirus involved in human disease in the Brazilian Amazon, using PRNT, which is the most specific serological test for detection of neutralizing antibodies for arboviruses. The PRNT is considered the gold standard for detecting neutralizing antibodies [49]. In addition, in the present study, we used a conservative criterion of seropositivity for PRNT, in which only samples with ≥90% reduction in plaque formation were considered seropositive. This criterion may appear conservative, but the main objective is to prevent the introduction of false positives in the data, even at cost of some false negatives.

The detection of neutralizing antibodies in patients of the state of Amapá suggests that OROV has circulated in the region during CHIKV introduction. Despite being highly specific, the PRNT does not allow us to discriminate time of exposure or sequential infections. It detects total neutralizing antibodies, and because of that, it is a useful serological method to indicate previous exposure. The difference in PRNT titers among serum samples may reflect different variables, which includes, beyond time of exposure and sequential infections, individual response and inoculum viral load. The time of exposure remains unknown, but the lower titers observed in some patients may indicate a long-date exposure, which is expected given the endemicity of oropouche fever in the Amazon region.

In the face of the biological effects of infectious diseases, the antibody function is the key information to understand the immune response to the infection, therapeutics strategy, and vaccine design and response. Assays that measure the antibody function give more information and differentiate from those that strictly measure the ability of an antibody to bind to its cognate antigen as ELISA and immunofluorescence. Neutralization capacity is one of the most desired antibody functions in scientific area, especially in infectious disease response. Opsonization, antibody-dependent cellular cytotoxicity (ADCC), and complement-mediated lysis of pathogens or of infected cells are other functions that antibodies can present [50]. There is no serological kit commercially available for OROV antibody detection. To date, PRNT is the most specific serological test for the differentiation of flavivirus infections, and is considered the reference method for detecting YF neutralizing antibodies and assessing the protective immune response following vaccination, according to the WHO [49,51]. A recent study assessed the intra- and inter-assay precisions, dilutability, specificity, and lower limit of quantification (LLOQ) using international standard YF serum and sera from vaccinees and human specimens collected through YF surveillance. The results showed that the PRNT assay showed 100% of intra-assay precision, 95.6% of inter-assay precision, 100% of specificity, 100% of LLOQ, and 95.3% of dilutability [52]. The main limitation in PRNT is that it cannot differentiate the isotype of neutralizing antibodies. IgG is the most abundant antibody isotype in the blood, followed by IgA and IgM [53,54]. Unfortunately, PRNT for CHIKV could not be done.

Enhancement of infection mediated by antibodies is observed in viruses such as DENV [55] and Ross River virus (*Ross River virus*, RRV) [56]. This phenomenon is characterized when non-neutralizing or sub-neutralizing antibodies bind to their epitope and, instead of neutralizing the viral particle; they facilitate its entry into the susceptible cell through surface Fc receptors [55,56,57]. Our samples had PRNT titers that range from 20 up to ≥640. To our knowledge, there is no report of the antibody-dependent facilitation of OROV infection. It has already been reported in the literature that peripheral blood mononuclear cell lines are susceptible to OROV infection, mainly Jurkat T. Monocytes and dendritic cells obtained from healthy donors are also susceptible to the virus, although they generate low yields of infectious particles and produce an intense antiviral response [58]. Despite being a virus of medical importance, studies on the pathogenesis and immune response generated by OROV infection are still scarce, and more studies are necessary on this subject.

It was believed that OROV was limited to the Amazon region, but the virus may be more dispersed than originally thought. In the state of Minas Gerais, located in the southeast region of Brazil, a strain belonging to genotype III of OROV was isolated from a non-human primate of the genus *Callithrix* in 2000 [59]. Ten years later, two patients living in São Paulo, also in the Southeast region, had OROV infection confirmed by indirect immunofluorescence [21]. Interestingly, one of them claimed to have traveled to the Amazon region (Rondônia) as expected. On the other hand, the other claimed to have traveled to the state of Bahia in the northeast. In another study carried out between 2011 and 2013, five patients with suspected dengue fever and eight pools of *Culex quinquefasciatus* mosquitoes from the state of Mato Grosso were positive for OROV by RT-PCR [20]. Such findings highlight the silent circulation of OROV, in addition to its ability to disperse and, consequently, its potential to spread through urban areas, showing the need for constant active surveillance. 

The emergence and re-emergence of arboviruses is associated with a set of factors related to the environment, vector and virus biology, and human activity. Uncontrolled urban growth and a lack of sanitation and water storage increase the number of breeding sites, as well as exposing humans to other pathogens [60]. For CHIKV, the urban cycle vectors are *Ae. aegypti* and *Ae. albopictus*. The first is a totally urban species with tropical and subtropical distribution, while the second is also found in temperate regions, having a wider range compared to *Ae. aegypti* [61,62]. High temperatures favor the presence of vectors in new areas and, consequently, increase the possibility of arbovirus establishment in new regions. Future climate change scenarios predict broader distribution of both species in the northern hemisphere [62]. The transmission of OROV in populated areas is believed to involve mostly the biting midges species *Culicoides paraensis*, which is found throughout most of Latin America and some U.S. states [63]. Some mosquito species are also known to act as an OROV vector, including *Culex quinquefasciatus*, *Coquillettidia venezuelensis*, *Mansonia venezuelensis*, and *Ae. serratus* [42,63].

## 5. Conclusions

The results obtained here corroborate the scenario reported in the state of Amapá in 2014–2015 when chikungunya epidemic emerged. Additionally, the present study has shown that some patients were also exposed, at some point, to OROV. Whether the OROV infection occurred prior or during the CHIKV circulation in the region is unknown, but because those arboviral infections share similar signs and symptoms, a silent circulation of enzootic arboviruses during introduction of exotic arboviruses may occur, and highlights the importance of syndromic cases’ surveillance to arboviruses in Brazil.

## Figures and Tables

**Table 1:** Chikungunya and Oropouche surveillance during an exanthematic outbreak occurring in 2014–2015 in Amapá, Amazon region, Brazil.

CHIKV	Cases/Total	(%)
Positive RNA detection	33/163	20.25
Recent CHIKV infection (anti-CHIKV IgM)	44/154	28.57
CHIKV exposure (anti-CHIKV-IgG)	19/151	12.58
**OROV**		
Positive RNA detection	0/166	
OROV exposure (NAb by PRNT_90_)	17/166	10.24

**Table 2:** Serological investigation of Oropouche-specific neutralizing antibodies by PRNT_90_ and Chikungunya Diagnosis using ELISA in 214 patients with acute febrile illness in the state of Amapá, 2014–2015.

Patient ID	Gender/Years-Old	Days of Illness	Municipality	OROV PRNT_90_ Titer	CHIKV Diagnosis		
					Anti-CHIKV IgM	Anti-CHIKV IgG	CHIKV RT-qPCR (Ct)
7 AP	Female, 41	NA	Santana	80	+	+	−
13 AP	Male, 36	17	Macapá	80	NT	NT	+ (29,45)
14 AP	Female, 56	NA	Macapá	80	−	−	−
26 AP	Male, 57	5	Oiapoque	20	+	UND	−
48 AP	Male, NA	7	Oiapoque	160	+	−	−
55 AP	Female, 38	6	Oiapoque	160	−	−	−
87 AP	Male, 37	NA	Santana	80	−	−	−
97 AP	Male, 24	NA	Macapá	40	+	−	−
103 AP	Female, 50	NA	Oiapoque	320	+	−	−
111 AP	Male, 60	NA	Santana	160	−	+	−
115 AP	Female, 62	NA	Macapá	≥640	+	+	−
140 AP	Female, 59	NA	Serra do Navio	40	+	−	−
166 AP	Female, 88	2	Oiapoque	80	−	UND	−

ID: identification; NA: not available, NT: not tested (sample unavailable for testing), UND: undetermined (sample was tested but its ratio was considered borderline according to test recommendations), Ct: cycle threshold, (+): positive, (−) negative.

## Data Availability

All the data available are included in the manuscript.
